# lncRNA H19 contributes to oxidative damage repair in the early age‐related cataract by regulating miR‐29a/TDG axis

**DOI:** 10.1111/jcmm.14489

**Published:** 2019-07-07

**Authors:** Tianyu Cheng, Mudong Xu, Bai Qin, Jian Wu, Yuanyuan Tu, Lihua Kang, Yong Wang, Huaijin Guan

**Affiliations:** ^1^ The Department of Ophthalmology Affiliated Hospital of Nantong University Nantong China; ^2^ Shanghai Jiao Tong University Affiliated Sixth People's Hospital Shanghai China

**Keywords:** early age‐related cataract, lncRNA H19, miRNA‐29a, TDG

## Abstract

Age‐related cataract (ARC) is caused by the exposure of the lens to UVB which promotes oxidative damage and cell death. This study aimed to explore the role of lncRNA H19 in oxidative damage repair in early ARC. lncRNAs sequencing technique was used to identify different lncRNAs in the lens of early ARC patients. Human lens epithelial cells (HLECs) were exposed to ultraviolet irradiation; and 8‐OHdG ELISA, Cell counting kit 8 (CCK8), EDU, flow cytometry and TUNEL assays were used to detect DNA damage, cell viability, proliferation and apoptosis. Luciferase assay was used to examine the interaction among H19, miR‐29a and thymine DNA glycosylase (TDG) 3'UTR. We found that lncRNA H19 and TDG were highly expressed while miR‐29a was down‐regulated in the three types of early ARC and HLECs exposed to ultraviolet irradiation, compared to respective controls. lncRNA H19 knockdown aggravated oxidative damage, reduced cell viability and proliferation, and promoted apoptosis in HLECs, while lncRNA H19 overexpression led to opposite effects in HLECs. Mechanistically, miR‐29a bound TDG 3'UTR to repress TDG expression. lncRNA H19 up‐regulated the expression of TDG by repressing miR‐29a because it acted as ceRNA through sponging miR‐29a. In conclusion, the interaction among lncRNA H19, miR‐29a and TDG is involved in early ARC. lncRNA H19 could be a useful marker of early ARC and oxidative damage repair pathway of lncRNA H19/miR‐29a/TDG may be a promising target for the treatment of ARC.

## 1 INTRODUCTION

With the trend of population ageing, age‐related cataract (ARC) has increasing incidence and remains the first cause of serious reversible visual impairment and blindness worldwide.^1^ The pathogenesis of ARC remains unclear but it is related to ultraviolet radiation, oxidative stress, metabolic disorders, drug‐induced changes, and other toxic factors.^2^ In particular, ultraviolet radiation mainly UVB is associated with the formation and development of ARC, since the energy of UVB can be absorbed by the lens and thus injure them.^3^, ^4^

Exposure of the lens to UVB can cause oxidative stress and reactive oxygen species (ROS). Oxidative stress and ROS‐induced DNA damage is considered as a key factor in the pathogenesis of ARC.^5^, ^6^ Ultraviolet radiation can cause DNA damage including modified bases, abasic sites and single‐strand breaks, and the DNA base damage is directly produced by ultraviolet (UV) photon absorption.^7^ The principal pathway to repair these bases is the base excision repair (BER) pathway, which consists of several DNA glycosylases, polymerases and ligases.^8^ Glycosylases such as human thymine DNA glycosylase (TDG) is the key player in the first step of base excision. TDG protects DNA from oxidation, deamination and conformational changes, and participates in Wnt signalling pathway to regulate the apoptosis and proliferation of cells.^9^, ^10^

lncRNA is a class of non‐coding RNA molecules that transcribe more than 200 nucleotides and could not encode a protein because of the lack of an effective open reading frame (ORF).^11^ Recently, several lncRNAs have been found to play a key role in ARC.^12^, ^13^, ^14^ In this study, we aimed to identify lncRNAs differentially expressed in ARC and elucidate their role in the pathogenesis of ARC. Using lncRNA sequencing technology, we found that lncRNA H19 was highly expressed in ARC. Furthermore, we revealed that lncRNA H19 acted as ceRNA through directly binding miR‐29a to regulate the expression of oxidative damage repair gene TDG.

## 2 MATERIALS AND METHODS

### 2.1 Clinical samples

Samples were obtained at the Affiliated Hospital of Nantong University between February 2017 and August 2018. According to Lens Opacities Classification System III (LOCS III),^15^ total 24 patients whose lenses with a score of C1‐C3, N1‐N3, or P1‐P3 including eight patients in each ARC category, and eight age‐matched controls who underwent vitrectomy operation of epiretinal membranes with a LOCSIII score of ≤C1, ≤N1 or ≤P1 were enrolled. We excluded patients with complex cataracts with high myopia, uveitis, ocular trauma or other known causes; other major eye diseases such as glaucoma, myopia, diabetic retinopathy and uveitis; and systemic diseases such as hypertension and diabetes. This study was conducted based on our protocols approved by the Ethics Committee of the Affiliated Hospital of Nantong University. All samples were collected after obtaining informed consent from the patients. Lens epithelium samples were dissected from the lens of patients during capsulotomy procedure and were easy to be identified because they were located in the anterior portion of the lens between the lens capsule and the lens fibres.

### 2.2 lncRNA sequence analysis

Total RNA from lens epithelium samples and cell lines were extracted using TRIzol reagent (Invitrogen, Carlsbad, CA) according to the manufacturer's instructions. RNA integrity and purity were verified by 1.5% agarose gel electrophoresis and OD260/280 between 1.8 and 2.0. The sequencing was performed at RiboBio (Guangzhou, China). The extracted RNA was subjected to high‐throughput sequencing, and the lncRNA database was analysed by DESeq to determine the differentially expressed lncRNAs.

### 2.3 Cell culture and UV irradiation

Human lens epithelial cell line SRA01/04 was purchased from Chinese Academy of Sciences (Shanghai, China), and cultured in DMEM (Gibco^®^ Invitrogen, Grand Island, NY) supplemented with 10% foetal bovine serum, 100 U/mL penicillin and 100 U/mL streptomycin at 37°C in a humidified incubator containing 5% CO2. SRA01/04 cells were divided into two groups, namely control group and experimental group. The experimental group cells were exposed to UVB light. The UVB lamps (XX‐15B, Spectroline, Westbury, NY) emit an energy spectrum of 280‐320 nm and a maximum emission peak at 312 nm. The intensity and dose of UBV were measured using a UVX Radiometer connected to a UVX31 Sensor (both were from UVP Inc, San Gabriel, CA).

### 2.4 Quantitative real‐time polymerase chain reaction

For quantitative real‐time polymerase chain reaction (RT‐qPCR) analysis, RNA was reverse‐transcribed by using PrimeScript RT reagent Kit with gDNA Eraser (Takara, Tokyo, Japan). For miRNA‐29a, Bulge‐Loop^TM^ miRNA RT primer (RiboBio, Guangzhou, China) was used. GADPH was used as the reference for H19 and TDG, and U6 was used as the reference for miRNA‐29a. The primers were as follows: H19 Forward TCTGAGAGATTCAAAGCCTCCAC, Reverse GTCTCCACAACTCCAACCAGTG; TDG Forward CAACAACTGATGGCTGAAGCTC, Reverse ACACTGCTATTCGTGGCTGA; GAPDH Forward CGGAGTCAACGGATTTGGTCGTAT, Reverse AGCCTTCTCCATGGTGGTGAAGAC. The relative abundance of RNA was calculated using the 2^−ΔΔCt^ method in Applied Biosystems PowerUp SYBR Green (Applied Biosystems, USA) on the ABI Step One Plus (Applied Biosystems, USA) according to the manufacturer's instructions.

### 2.5 RNA fluorescence in situ hybridization

Fluorescent in Situ Hybridization Kit (RiboBio, Guangzhou, China) was used to identify cellular location of lncRNA H19. First, SRA01/04 cells were fixed with 4% PFA fixation solution for 15 minutes. Then the cells were permeabilized in PBS containing 0.5% Triton X‐100 for 30 minutes at 4°C and pre‐hybridized in pre‐hybridization solution for 30 minutes at 37°C. Next, fluorescence in situ hybridization (FISH) probes were added to the hybridization solution and incubated overnight at 37°C in the dark. The next day, the cells were counterstained with DAPI and then examined under confocal microscopy (Leica, Germany).

### 2.6 Cell transfection

SRA01/04 cells were cultured in 6‐well plates to 60%‐70% confluence prior to transfection. Si‐H19, si‐H19 NC, si‐TDG, si‐TDG NC, miRNA‐29a mimic and mimic control were purchased from RiboBio (Guangzhou, China). H19 cDNA was amplified and cloned into pcDNA vector (Hanyinbt, Shanghai, China). The siRNA or vector was transfected into cells with lipofectamine 3000 transfection reagent (Invitrogen, USA) according to the manufacturer's protocol.

### 2.7 8‐OHdG ELISA

Absolute levels of 8‐OHdG were measured using the Human 8‐OHDG ELISA Kit Assay (Ameko, Shanghai, China). Briefly, SRA01/04 cells were seeded onto 96‐well plates and then treated under different conditions. At the appropriate time, the supernatant was collected for ELISA assay. The absorbance was measured at 450 nm and the protein levels of 8‐OHdG were calculated by comparing the OD of the samples to the standard curve.

### 2.8 CCK8 and EDU assay

Cell counting kit 8 assay (Dojindo Laboratory, Kumamoto, Japan) and EdU assay (RiboBio, Guangzhou, China) were used to detect cell viability and proliferation. For CCK8 assay, SRA01/04 cells were seeded in 96‐well plates and cultured for indicated time. Then 10 μL of CCK8 solution was added and the absorbance at 450 nm was measured by a microplate reader (BioTek, Vermont). The cell viability was then calculated. For EdU assay, SRA01/04 cells were incubated with EdU according to the manufacturer's protocol and then fixed with 4% PFA fixation solution. The cells were stained with 1 × Apollo reaction cocktail for 30 minutes before being incubated with Hoechst 33342 for 30 minutes. The cells were then visualized under a fluorescence microscope (Leica, Germany).

### 2.9 Flow cytometry

SRA01/04 cells were incubated with FITC and PI using the Annexin V‐FITC/PI Apoptosis Detection kit (BD, Franklin Lakes, NJ) at room temperature for 15 minutes in the dark and the samples were analysed by flow cytometry (BD, New Jersey). Cells showing only Annexin V‐FITC‐positive staining were considered to be early apoptosis, while cells stained with both Annexin V‐FITC and PI were considered to be late apoptosis.

### 2.10 Tunel assay

SRA01/04 cells were seeded into 24‐well plates with glass coverslips. After various treatments, cells were fixed with 4% PFA fixation solution for 30 minutes at room temperature. The coverslips were washed in 0.01% PBS for three times and incubated in permeabilization solution for 2 minutes on ice. Then the coverslips were incubated with TUNEL mixture (Roche, Basel, Switzerland) for 1 hour in a dark humidity chamber at 37°C. After washing with PBS, the coverslips were incubated with Hoechst 33258 (RiboBio, Guangzhou, China) for 10 minutes and then examined under a fluorescence microscope (Leica, Germany).

### 2.11 Western blot analysis

SRA01/04 cells were collected and lysed in RIPA containing phenylmethanesulphonyl fluoride (Beytime, Shanghai, China) at 4°C for 30 minutes. Protein extracts were separated on 10% SDS‐PAGE gels and then transferred onto PVDF membranes (Thermo Fisher Scientific, Pittsburgh, PA). After blocking in TBST with 5% non‐fat milk for 2 hours at 37°C, membranes were probed with the primary rabbit monoclonal antibodies against TDG (1:1,000 dilution, Abcam) and GADPH (1:1,000 dilution, Abclonal) overnight at 4°C. After incubation with secondary peroxidase‐conjugated AffiniPure Goat Anti‐rabbit IgG (1:8,000 dilution, Jackson ImmunoResearch), the membranes were exposed to light film (BioMax MR; Kodak, Rochester, NY). The grey value of each protein band was measured, and the TDG protein expression was normalized to GAPDH levels.

### 2.12 Luciferase activity assay

The wild‐type or mutant sequences of the H19 fragment and the 3'UTR of TDG containing the binding sites with miR‐29a were cloned into a pmirGLO Dual‐luciferase Vector (Hanyinbt, Shanghai, China). For luciferase reporter assay, miR‐29a mimics or the negative control (NC) was co‐transfected with H19‐WT or H19‐MUT into SRA01/04 cells. After 24 hours, the relative luciferase activities were determined using the Dual‐Luciferase Reporter Assay System (E1910, Promega, Madison, WI) following the manufacturer's instructions.

### 2.13 Statistical analysis

Statistical analysis was performed with spss 16.0 software (SPSS Inc, USA). All experiments were repeated three times, and all data were presented as mean ± SD. The differences between groups were assessed by Student's *t* test, and *P* < 0.05 was considered significant.

## 3 RESULTS

### 3.1 Up‐regulation of lncRNA H19 in the lens of ARC patients and HLECs exposed to UVB

**Figure 1:**
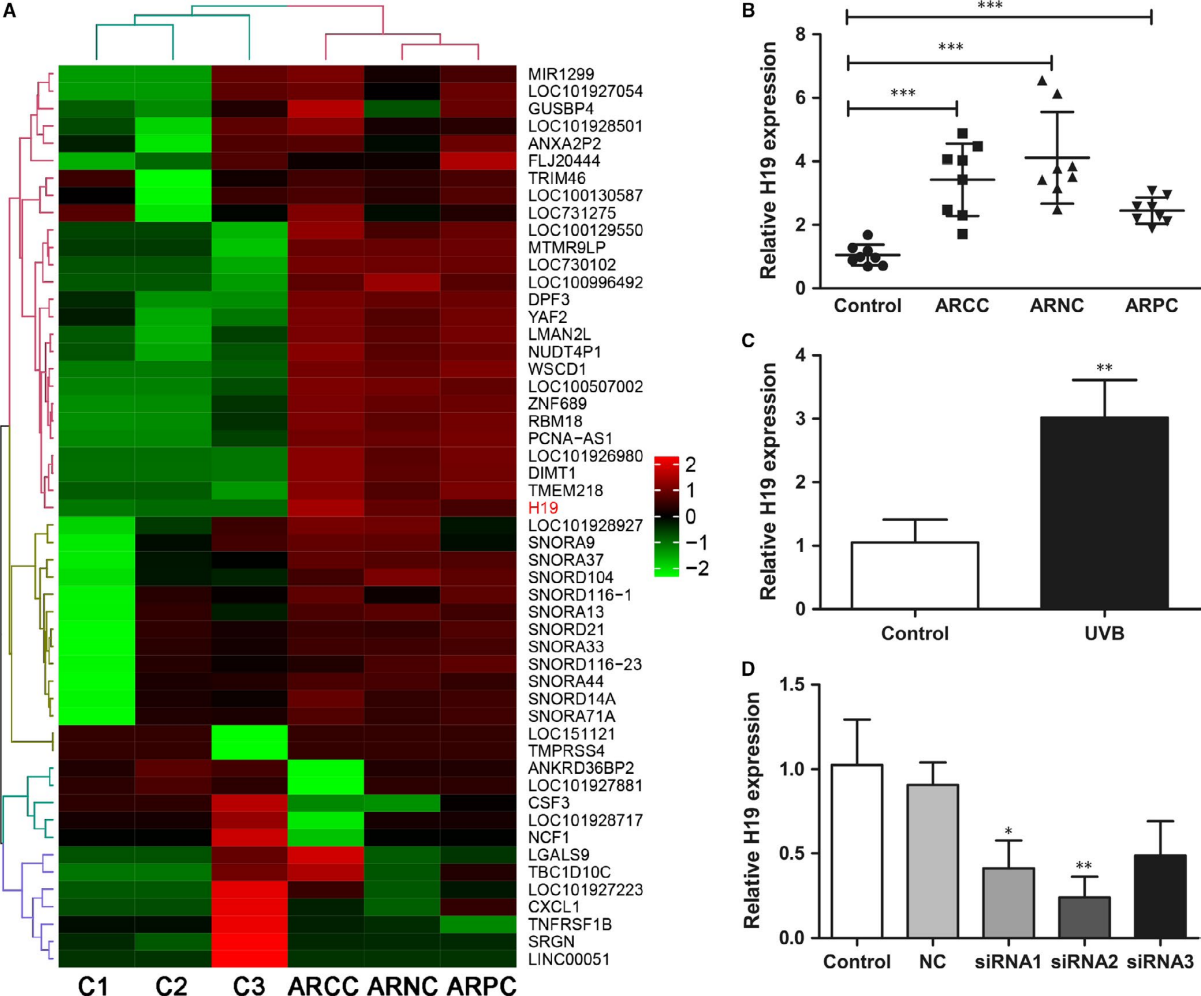
lncRNA H19 is up‐regulated in the lens of age‐related cataract (ARC) patients in human lens epithelial cells (HLECs) exposed to UVB irradiation. (A), Heat maps were generated from hierarchical cluster analysis to show differentially expressed lncRNAs between control and early ARC lens samples. The colour scale illustrated the relative expression level of lncRNAs across different samples. Red indicated up‐regulation, while green indicated down‐regulation. C1‐C3 indicated three controls. ARCC, age‐related cortical cataract; ARNC, age‐related nuclear cataract; ARPC, age‐related posterior subcapsular cataract. (B), lncRNA H19 expression levels were significantly high in the three types of the early ARC compared to controls, ***P < 0.001. (C), lncRNA H19 expression levels were increased significantly in HLECs exposed to UVB. **P < 0.01. (D) lncRNA H19 expression levels in HLECs transfected with H19 siRNAs. *P < 0.05, **P < 0.01

To identify different lncRNAs in the lens between control and early ARC patient, we performed lncRNAs sequencing. We set the threshold as the fold change >2.0, and identified 52 differentially expressed lncRNAs, including 10 down‐regulated lncRNAs and 42 up‐regulated lncRNAs (Figure 1A). We selected lncRNA H19 because it showed high level of up‐regulation of expression in ARC patients. Real‐time PCR confirmed that lncRNA H19 level was significantly up‐regulated in the three types of early ARC compared with the controls (Figure 1B). In addition, we found that expression levels of lncRNA H19 in SRA01/04 cells were increased significantly after UVB irradiation (Figure 1C).

In order to reveal the role of lncRNA H19 in UVB‐induced damage in HLECs, we transfected si‐H19 and pcDNA‐H19 into SRA01/04 cells to down‐regulate or up‐regulate lncRNA H19 levels. Real‐time PCR showed that the expression of lncRNA H19 was reduced significantly in cells transfected with H19‐siRNAs, especially in cells transfected with H‐19‐siRNA2 (Figure 1D). Thus we chose H19‐siRNA2 for the following experiments.

### 3.2 Interference of lncRNA H19 levels regulates DNA damage, viability, proliferation and apoptosis in HLECs exposed to UVB

**Figure 2:**
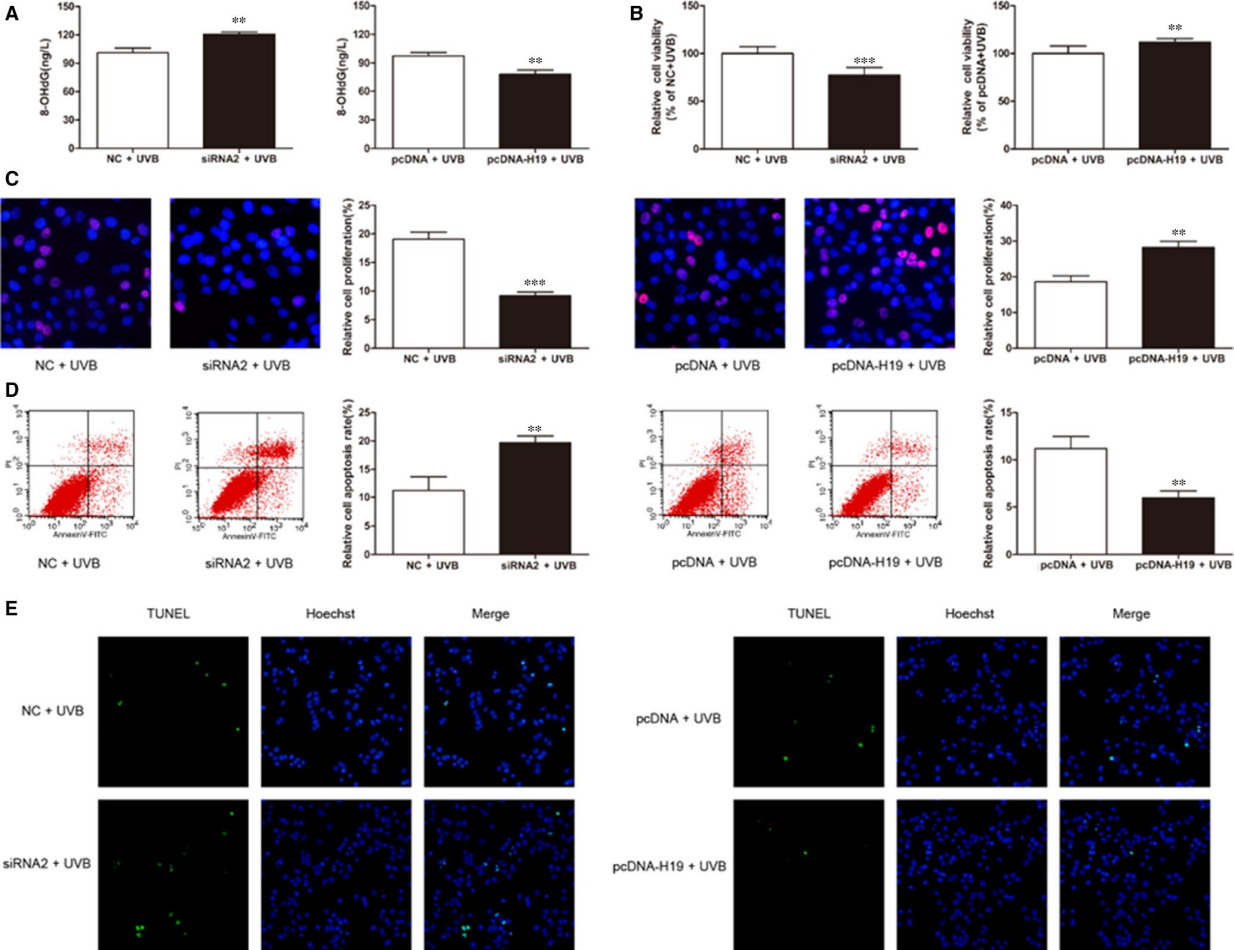
Knockdown and overexpression of lncRNA H19 regulate the function of human lens epithelial cells (HLECs). (A), Oxidative damage was detected by 8‐OHdG ELISA assay, **P < 0.01. (B), Cell viability was detected by CCK8 assay, **P < 0.01, *P < 0.05. (C), Cell proliferation was analysed by EdU assay, ***P < 0.001, **P < 0.01. (D), Apoptotic cells were analysed by flow cytometry, **P < 0.01. (E), TUNEL assay of apoptotic cells

Next we transfected HLECs with H‐19‐siRNA2 and pcDNA‐H19 to down‐regulate and up‐regulate H19 levels respectively. Based on 8‐OHdG ELISA assay, we found that lncRNA H19 knockdown aggravated oxidative damage, while overexpression of lncRNA H19 alleviated oxidative damage in SRA01/04 cells (Figure 2A). CCK8 and EdU assay showed that the viability and proliferation of SRA01/04 cells significantly reduced after lncRNA H19 knockdown, but significantly increased after lncRNA H19 overexpression (Figure 2B and 2). In addition, flow cytometry showed that apoptotic SRA01/04 cells significantly increased after lncRNA H19 knockdown, but significantly decreased after lncRNA H19 overexpression, compared with the respective control group (Figure 2D). TUNEL assay showed similar results of apoptosis in SRA01/04 cells (Figure 2E).

### 3.3 lncRNA H19 directly binds miR‐29a and represses its expression in HLECs

**Figure 3:**
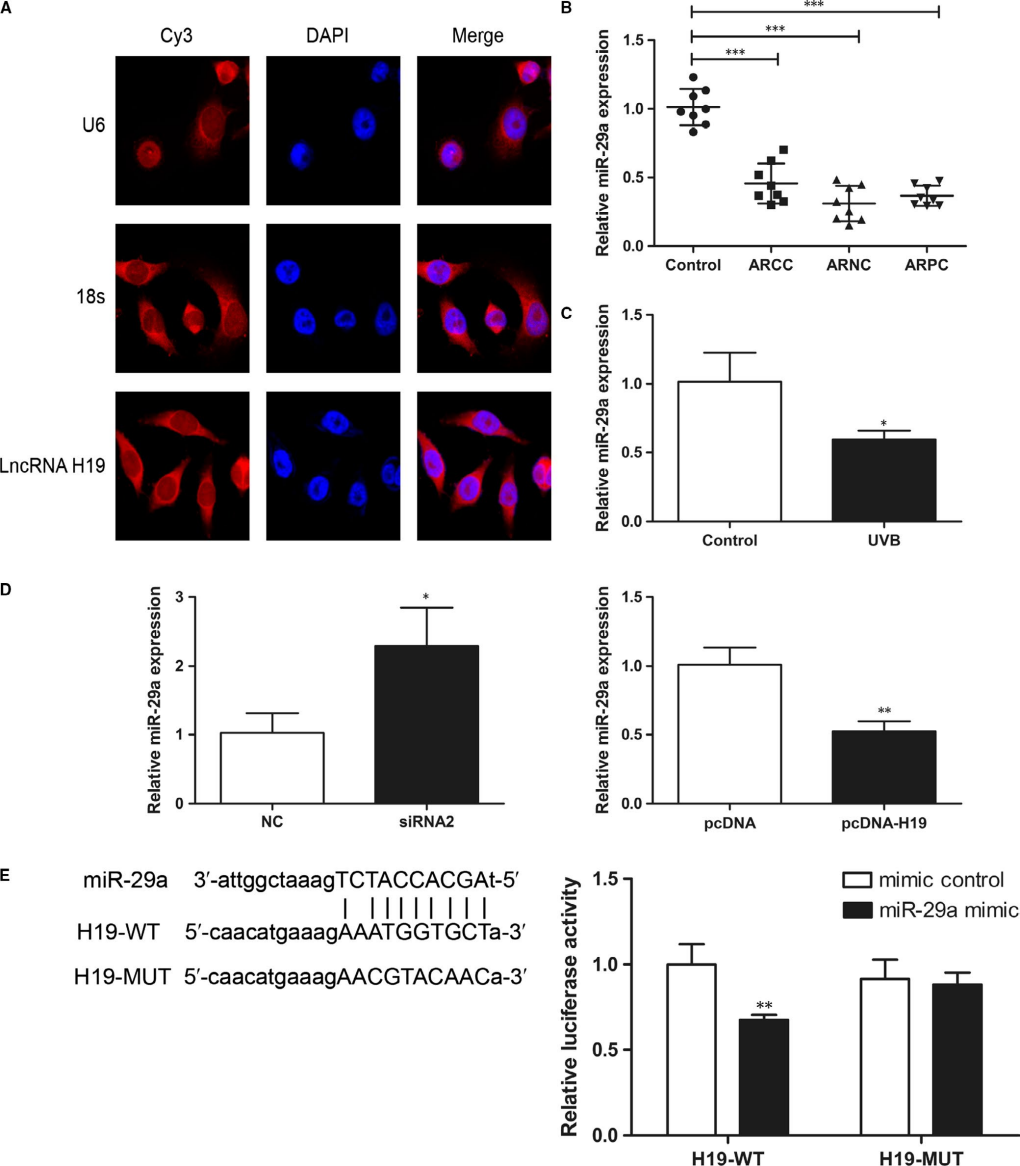
lncRNA H19 inhibits miR‐29a in human lens epithelial cells (HLECs). (A), lncRNA H19 was enriched significantly in the cytoplasm by FISH assay. (B), miR‐29a expression was declined in the three types of early ARC. ***P < 0.001. (C), miR‐29a expression decreased significantly in HLECs exposed to UVB. *P < 0.05. (D), miR‐29a expression increased in HLECs transfected with si‐H19 and decreased in the cells transfected with pcDNA‐H19. *P < 0.05, **P < 0.01. (E), The luciferase activity of H19‐WT but not H19‐MUT was inhibited by miR‐29a mimic. **P < 0.01

Fluorescence in situ hybridization assay showed that lncRNA H19 was significantly enriched in the cytoplasm of SRA01/04 cells compared to the nucleus (Figure 3A). Therefore, we proposed that lncRNA H19 may function as ceRNA, and miR‐29a was predicted as a competing miRNA of lncRNA H19 by starbase 2.0. Interestingly, miR‐29a level dramatically decreased in cataractous rat LECs.^16^ Indeed, we found that miR‐29a expression was reduced in the three types of early ARC compared to controls (Figure 3B). Moreover, miR‐29a expression was significantly decreased in SRA01/04 cells exposed to UVB compared to control cells (Figure 3C). Furthermore, RT‐PCR analysis showed that miR‐29a levels were increased in SRA01/04 cells transfected with H19 siRNA2, and decreased in cells transfected with pcDNA‐H19 (Figure 3D). These results demonstrate the negative correlation of lncRNA H19 and miR‐29a levels. In addition, dual‐luciferase assay confirmed that lncRNA H19 inhibited miR‐29a expression by directly sponging miR‐29a (Figure 3E).

### 3.4 miR‐29a directly binds TDG 3'UTR and lncRNA H19 up‐regulates TDG expression in HLECs

**Figure 4:**
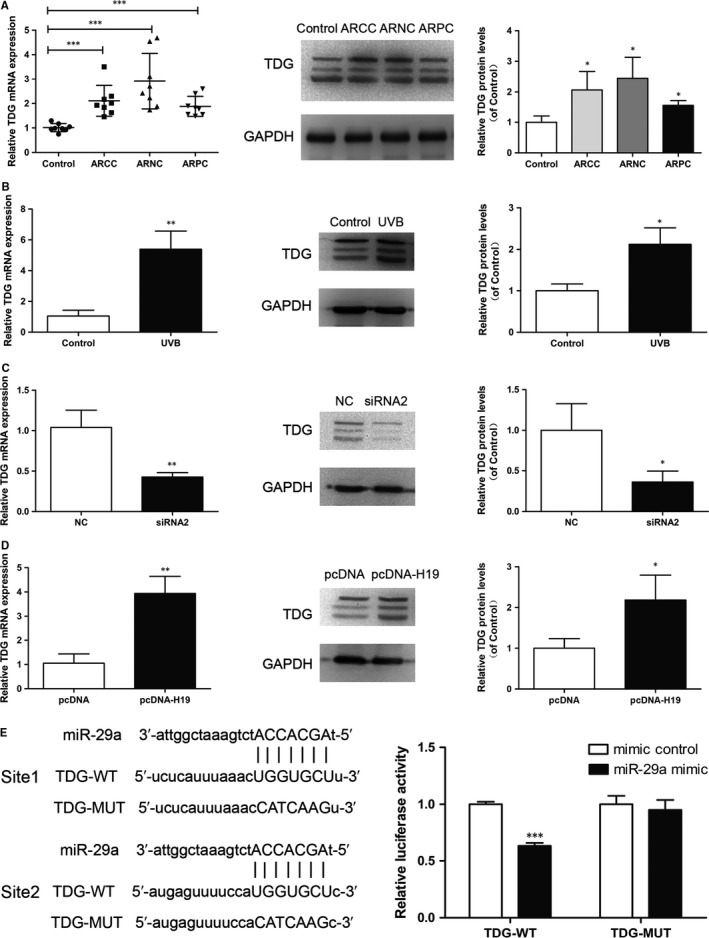
Thymine DNA glycosylase (TDG) is up‐regulated in the lens of age‐related cataract (ARC) patients in human lens epithelial cells (HLECs) exposed to UVB irradiation. (A), TDG mRNA and protein levels increased in lens of early ARC, ***P < 0.001, *P < 0.05. (B), TDG mRNA and protein levels increased in HLECs exposed to UVB, **P < 0.01, *P < 0.05. (C), TDG mRNA and protein levels decreased in HLECs transfected with si‐H19, **P < 0.01, *P < 0.05. (D), TDG mRNA and protein levels increased in HLECs transfected with pcDNA‐H19, **P < 0.01, *P < 0.05. (E), The luciferase activity of TDG 3'UTR‐WT but not TDG 3'UTR‐MUT was inhibited by miR‐29a mimic. ***P < 0.001

Next we used targetscanhuman 7.2 to predict downstream oxidative damage repair gene. Interestingly, TDG 3'UTR contains two miR‐29a binding sites, and previous study showed that miR‐29a mimics decreased TDG mRNA while miR‐29a inhibitors increased TDG mRNA.^17^ Therefore, we focused on oxidative damage repair gene TDG as the downstream gene. TDG mRNA and protein levels were significantly higher in early ARC and SRA01/04 cells exposed to UVB compared to controls (Figure 4A and B). In addition, TDG mRNA and protein levels were decreased in SRA01/04 cells transfected with H19 siRNA2, and increased in cells transfected with pcDNA‐H19 (Figure 4C and D). To further verify the binding between TDG 3'UTR and miR‐29a, we mutated miR‐29a binding sites in TDG 3'UTR, and dual‐luciferase reporter assay showed that miR‐29a could only bind wild‐type TDG3'UTR (Figure 4E).

### 3.5 lncRNA H19 up‐regulates TDG expression by repressing miR‐29a

**Figure 5:**
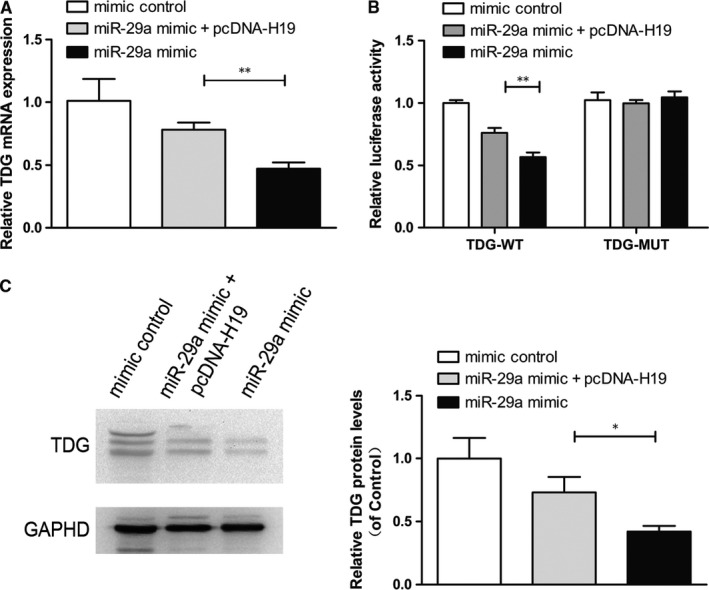
lncRNA H19 up‐regulates the expression of thymine DNA glycosylase (TDG) by repressing miR‐29a. (A), TDG mRNA expression decreased in HLECs transfected with miR‐29a mimic, and increased in cells transfected with pcDNA‐H19. **P < 0.01. (B), Overexpression of lncRNA H19 restored luciferase activity of TDG‐WT suppressed by miR‐29a compared with NC. **P < 0.01. (C), Western blot analysis showed that lncRNA H19 overexpression recovered TDG protein level

To further verify whether lncRNA H19 increases TDG expression by repressing miR‐29a, we co‐transfected miR‐29a mimics or NC, pcDNA‐H19 or pcDNA into SRA01/04 cells. RT‐PCR showed that overexpression of lncRNA H19 increased TDG mRNA expression compared with cells only transfected with miR‐29a (Figure 5A). Dual‐luciferase reporter assay demonstrated that lncRNA H19 overexpression could rescue luciferase activity of TDG‐WT suppressed by miR‐29a (Figure 5B). Moreover, the overexpression of lncRNA H19 increased TDG protein expression compared with cells only transfected with miR‐29a (Figure 5C).

## 4 DISCUSSION

Recent studies reported that lncRNAs were widely expressed in ARC, which may promote or inhibit the occurrence and development of ARC.^12^, ^13^, ^14^ In this study, we focused on early ARC and explored the oxidative damage repair mechanism in order to prevent the development of early ARC. Our results demonstrate that lncRNA H19 was up‐regulated in the anterior lens capsules of the early development ARC and UVB irradiation‐induced oxidative damage model cells.

lncRNA H19 is located on human chromosome 11 p15.5 and participates in the regulation of different biological processes by acting as a ceRNA that released the miRNA's targets via the competition for miRNA to influence the related protein factors.^18^ MiRNAs are short RNA sequences ranging from 19 to 23 nt which are mainly paired with complementary target mRNA in 3'‐UTR to inhibit the mRNA translation or promote the mRNA degradation. The correlation between lncRNA, miRNA and mRNA suggests a complicated regulatory mechanism in diverse diseases.^19^

Notably, several miRNAs are specifically expressed in the lens, suggesting their important role in regulating HLECs function, such as let‐7b, miR‐34a, miRNA‐125b.^20^, ^21^, ^22^, ^23^ Previous study showed that miR‐29a level dramatically decreased in cataractous LECs from Shumiya Cataract Rats.^16^ In this study, we found that miR‐29a expression decreased in the three types of early ARC and in HLECs exposed to UVB irradiation. These results suggest that down‐regulation of miR‐29a is implicated in the development of ARC.

Oxidative stress and ROS accumulation can induce DNA damage and lead to the development of ARC.^24^, ^25^ TDG plays an important role in aberrant BER pathway of oxidatively damaged DNA.^26^ In this study, we found that TDG was highly expressed in the early ARC and in HLECs exposed to UVB irradiation. Furthermore, we demonstrated that lncRNA H19 up‐regulated the expression of TDG by sponging miR‐29a in HLECs, because miR‐29a could directly bind TDG 3'UTR to repress TDG expression. These data indicate that lncRNA H19 plays a role in early ARC through regulating oxidative damage repair pathway.

In conclusion, lncRNA H19 is up‐regulated in the lens of ARC patients, and its knockdown can aggravate UVB irradiation‐induced oxidative damage, reduce cells viability and proliferation, and increase apoptosis of HLECs. In addition, for the first, we elucidated the interaction among lncRNA H19, miR‐29a and TDG in early ARC. These results suggest that lncRNA H19 could be a useful marker of early ARC and the oxidative damage repair pathway of lncRNA H19/miR‐29a/TDG may be a promising target for the treatment of ARC.

## CONFLICT OF INTEREST

The authors declare no conflict of interest.

## AUTHOR'S CONTRIBUTION

Tianyu Cheng, Mudong Xu, Bai Qin, Jian Wu, Yuanyuan Tu, Lihua Kang and Yong Wang performed the experiments and analysed the data. Huaijin Guan designed the study.

## Data Availability

All data are available upon reasonable request.
